# Ecthyma gangrenosum without bacteremia in a previously healthy man: a case report

**DOI:** 10.1186/1752-1947-2-14

**Published:** 2008-01-22

**Authors:** Serap Gençer, Serdar Özer, Aylin Ege Gül, Mustafa Doğan, Öznur Ak

**Affiliations:** 1 Department of Infectious Diseases and Clinical Microbiology, Kartal Dr. Lütfi Kırdar Training and Research Hospital, Istanbul, Turkey; 2 Department of Pathology, Kartal Dr. Lütfi Kırdar Training and Research Hospital, Istanbul, Turkey

## Abstract

**Introduction:**

Ecthyma gangrenosum is known as a characteristic lesion of *Pseudomonas aeruginosa *sepsis and is usually seen in immunocompromised patients.

**Case presentation:**

A previously healthy 63-year-old man was admitted with sloughy necrotic ulcerations of the skin over his sternum. He was afebrile and in good condition. A skin biopsy revealed ecthyma gangrenosum. Blood cultures remained sterile, but a culture of biopsy material grew *Pseudomonas aeruginosa*.

**Conclusion:**

Ecthyma gangrenosum may develop even in the absence of bacteremia and even in immunocompetent patients. It should be considered as a possible diagnosis even when a previously healthy patient has negative blood cultures.

## Introduction

Ecthyma gangrenosum is a characteristic necrotic and bullous skin lesion known to be caused by *Pseudomonas aeruginosa *sepsis. It is usually seen in immunocompromised people. However, it is rare to see ecthyma gangrenosum in people with no evidence of bacteremia [1-6] and in those who were previously healthy [7]. Herein, we report a rare presentation of ecthyma gangrenosum in a previously healthy adult male without bacteremia.

## Case presentation

A previously healthy 63-year-old man was admitted with a two week history of wounds over his sternum. Twenty days before hospital admission, he had developed fever and sore throat. Oral amoxicillin was given. Six days later he noted an erythematous skin lesion over his sternum. One week later his sore throat resolved but the skin lesion worsened. He presented to a dermatologist and was diagnosed with erysipelas and received oral amoxicillin again. Over the next two days, the skin lesion evolved to form vesicles that were drained in the emergency department of another hospital. The lesion progressed to ulceration over the next five days.

The patient had no history of immunosuppressive disease or treatment.

**Figure 1:**
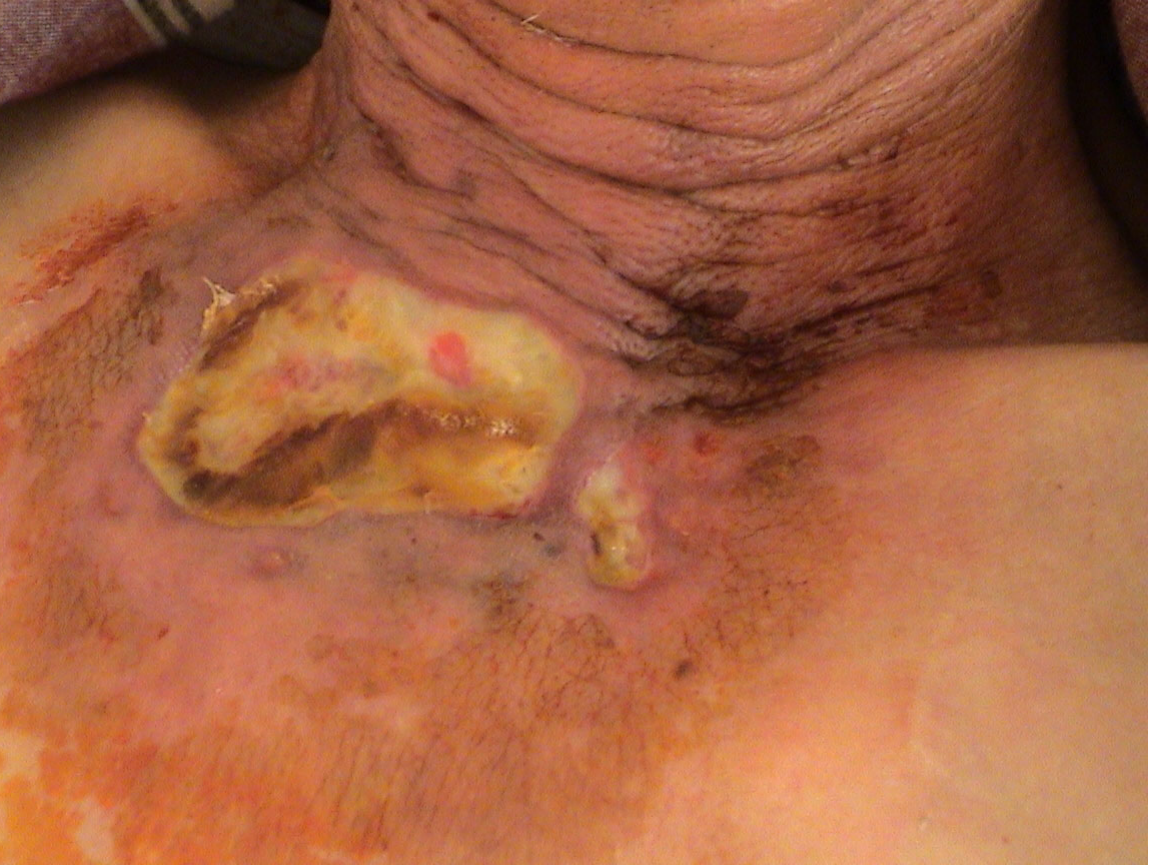
Sloughy, necrotic ulceration of the skin characteristic of ecthyma gangrenosum.

On admission he was afebrile (temperature of 37,2°C), and hemodynamically stable. Physical examination revealed two sloughy necrotic ulcerations of the skin over the sternum with hyperemic margins. These lesions measured about 6 × 4 cm. and 2 × 1 cm. (Figure 1). The white blood cell count was 12.700/mm^3^, the hemoglobin level was 10.8 g/dL, and the platelet count was 561.000/mm^3^. The erythrocyte sedimentation rate was 78 mm/hour, the serum C-reactive protein level was 5,6 mg/L (range 0 – 5 mg/L). Liver function tests, blood urea nitrogen and serum creatinine levels and chest radiographs were normal.

**Figure 2:**
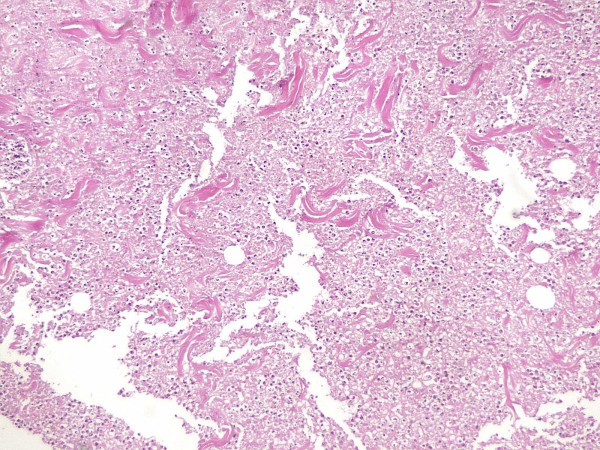
Ulcerated inflamatory cell infiltration, vascular proliferation and wide necrosis characteristic of ecthyma gangrenosum.

Blood cultures were taken. A skin biopsy with culture was performed. While the results of cultures were pending, antibiotic therapy with imipenem (500 mg. IV every 6 hours) was initiated. Pathological examination of the biopsy material revealed ulcerated inflammatory cell infiltration, vascular proliferation and wide necrosis characteristic of ecthyma gangrenosum (Figure 2). Blood cultures remained sterile, but a culture of the biopsy material grew *Pseudomonas aeruginosa*. Antibiotic treatment was continued for 14 days, after which the patient was admitted to the Department of Plastic Surgery for skin grafting. Two months later, the skin lesion had healed. One year later, he remains healthy.

## Discussion

It is well known that ecthyma gangrenosum is one of the major dermatologic manifestations of severe, systemic *Pseudomonas aeruginosa *infection. It occurs in only 1–6% of patients with Pseudomonas bacteremia [8]. It had been considered to be pathognomonic of Pseudomonas sepsis until it was described in cases with infections caused by group A Streptococcus, *Aeromonas hydrophila, Staphylococcus aureus, Serratia marcescens, Pseudomonas maltophilia, Escherichia coli, Candida albicans, Aspergillus *species and *Mucor *species [9].

Ecthyma gangrenosum is usually seen in immunocompromised patients with leukemia, lymphoma, other malignant diseases, severe burns or organ transplant, or in people receiving immunosuppressive therapy [1,2,4,6]. However, it has been reported also in patients without previously identified medical problems. Most of them had a concurrent viral infection or had received recent antibiotic therapy [7]. Ecthyma gangrenosum might be the first manifestation of an underlying medical problem and previously healthy patients should be followed closely in the future [7,10].

The lesion begins as a painless red macule that enlarges and becomes a slightly elevated papule. It evolves to a hemorrhagic bulla that ruptures, forming a gangrenous ulcer with a gray-black eschar surrounded by an erythematous halo [1]. Classically, the pathogen is isolated from the skin lesions as well as from the blood. These lesions may occur anywhere, but are most usual on the anogenital region, buttocks, extremities, abdomen, axillae and rarely on the face [1,5].

Histologically, the lesions represent a necrotising vasculitis caused by direct bacterial invasion of the media and adventitia of the vascular walls, but not the intima [2]. In general, acute mixed inflammatory cell infiltration and vascular proliferation are seen in the dermis, often involving the subcutaneous tissue. Elastases produced by Pseudomonas destroy the elastic small vessels, leading to hemorrhage and release of organisms into the surrounding tissue. Protease and endotoxin A elaborated by bacilli are responsible for the direct tissue destruction and ulcerative lesions. [5,9].

In classic bacteremic ecthyma gangrenosum, the lesion represents a blood-borne metastatic seeding of *Pseudomonas aeruginosa *to the skin. However, there are a few reports that ecthyma gangrenosum can represent localized skin eruptions that are not accompanied by bacteremia or systemic infection [1-6].

The source of infection in this patient cannot be determined with certainty, but it is possible that the patient presented with erysipelas which subsequently became colonized and superinfected with hospital-acquired *Pseudomonas aeruginosa *while draining and then developed into ecthyma gangrenosum. Negative blood cultures suggest that ecthyma gangrenosum occurred as a primary lesion at a site of prior skin trauma.

Early diagnosis and aggressive therapy are important in the management of ecthyma gangrenosum. An antipseudomonal beta-lactam antibiotic with or without an aminoglycoside is appropriate for treatment of both bacteremic and nonbacteremic ecthyma gangrenosum [5]. The absence of bacteremia is associated with the best outcome. Patients with Pseudomonas bacteremia have been reported to have a mortality rate of 38% [7]. On the other hand, only two patients (15%) died in a review of 13 patients with ecthyma gangrenosum without bacteremia [1]. In another study, the mortality rate was 7.5 % in the group of patients with skin lesions considered to be primary and 20 % in the group of patients with skin lesions considered to be secondary to bacteremia [3].

## Conclusion

As we point out in this case, ecthyma gangrenosum may develop even in the absence of bacteremia and even in immunocompetent people. It may be treated with appropriate antibiotics upon diagnosis by tissue culture and microscopic examination. In conclusion, ecthyma gangrenosum should be considered as a possible diagnosis even when a previously healthy patient has negative blood cultures.

## Competing interests

The author(s) declare that they have no competing interests.

## Authors' contributions

SG participated in patient management, diagnosis, reviewed the literature and drafted the manuscript. SÖ, MD and ÖA participated in patient management and diagnosis. AE made the pathological examination and diagnosis. All authors read and approved the final manuscript.

## Consent

Written informed consent was obtained from the patient for publication of this case report and any accompanying images. A copy of the written consent is available for review by the Editor-in-Chief of this journal.
